# Pediatric Celiac Disease Patients Show Alterations of Dendritic Cell Shape and Actin Rearrangement

**DOI:** 10.3390/ijms22052708

**Published:** 2021-03-08

**Authors:** Valentina Discepolo, Giuliana Lania, Maria Leonarda Gertrude Ten Eikelder, Merlin Nanayakkara, Leandra Sepe, Rossella Tufano, Riccardo Troncone, Salvatore Auricchio, Renata Auricchio, Giovanni Paolella, Maria Vittoria Barone

**Affiliations:** 1 European Laboratory for the Investigation of Food Induced Diseases, Department of Translational Medical Science, Section of Pediatrics, and ELFID, University Federico II, Via S. Pansini 5, 80131 Naples, Italy; valentina.discepolo@unina.it (V.D.); giuliana.lania@gmail.com (G.L.); merlinnanayakkara@gmail.com (M.N.); troncone@unina.it (R.T.); salauric@unina.it (S.A.); rauricchio@unina.it (R.A.); giovanni.paolella@unina.it (G.P.); 2 Department of Gynecology, Leiden University Medical Centre, Albinusdreef 2, 2333 ZA Leiden, The Netherlands; m.teneikelder@gmail.com; 3 Department of Molecular Medicine and Medical Biotechnologies, University of Naples Federico II, Via S. Pansini 5, 80131 Naples, Italy; sepe@ceinge.unina.it (L.S.); rossella.tufano@unina.it (R.T.)

**Keywords:** celiac disease, dendritic cells, actin cytoskeleton, cell shape, adhesion, fibronectin, RhoA, ARHGAP31

## Abstract

Celiac disease (CD) is a frequent intestinal inflammatory disease occurring in genetically susceptible individuals upon gluten ingestion. Recent studies point to a role in CD for genes involved in cell shape, adhesion and actin rearrangements, including a Rho family regulator, Rho GTPase-activating protein 31 (ARHGAP31). In this study, we investigated the morphology and actin cytoskeletons of peripheral monocyte-derived dendritic cells (DCs) from children with CD and controls when in contact with a physiological substrate, fibronectin. DCs were generated from peripheral blood monocytes of pediatric CD patients and controls. After adhesion on fibronectin, DCs showed a higher number of protrusions and a more elongated shape in CD patients compared with controls, as assessed by immunofluorescence actin staining, transmitted light staining and video time-lapse microscopy. These alterations did not depend on active intestinal inflammation associated with gluten consumption and were specific to CD, since they were not found in subjects affected by other intestinal inflammatory conditions. The elongated morphology was not a result of differences in DC activation or maturation status, and did not depend on the human leukocyte antigen (HLA)-DQ2 haplotype. Notably, we found that ARH-GAP31 mRNA levels were decreased while RhoA-GTP activity was increased in CD DCs, pointing to an impairment of the Rho pathway in CD cells. Accordingly, Rho inhibition was able to prevent the cytoskeleton rearrangements leading to the elongated morphology of celiac DCs upon adhesion on fibronectin, confirming the role of this pathway in the observed phenotype. In conclusion, adhesion on fibronectin discriminated CD from the controls’ DCs, revealing a gluten-independent CD-specific cellular phenotype related to DC shape and regulated by RhoA activity.

## 1. Introduction

Celiac disease (CD) is characterized by activation of both the adaptive and the innate immune responses upon gluten ingestion. Some gliadin peptides (i.e., A-gliadin P57-68) are presented by antigen-presenting cells, in the context of human leukocyte antigen (HLA)-DQ2 and/or -DQ8 molecules after deamidation by the enzyme tissue transglutaminase (tTg). The presentation of these peptides by antigen-presenting cells, such as dendritic cells (DCs), is able to induce a gluten-specific adaptive T helper (Th)-1 inflammatory response [1]. Other gliadin peptides (i.e., P31–43) are able to initiate an innate immune response [2,3]. The intestinal mucosal damage typical of CD is characterized by infiltration of intraepithelial lymphocytes, proliferation of crypt enterocytes as an early alteration of CD mucosa resulting in crypt hyperplasia [4], and ultimately villous atrophy mediated by cytotoxic T cells and inflammatory cytokines. These alterations are reverted upon gluten withdrawal.

Modifications of the actin cytoskeleton [5] and gluten-dependent shortening of enterocytes have been found in CD patients [6]. Alterations of the tight junctions (TJ) regulating intestinal permeability persisted even in patients on a gluten-free diet (GFD) with a normalized intestinal architecture [7,8,9,10,11]. Moreover, a disruption of the intestinal epithelium integrity was found in early-stage CD [12]. Polymorphisms in the TJ genes Par-3 family cell polarity regulator (PARD3) and membrane-associated guanylate kinase, WW and PDZ domain-containing 2 (MAGI2) have been associated with CD susceptibility [13]. Interestingly, the protein phosphatase 2 regulatory subunit B alpha (PPP2R3A) gene, implicated in the negative control of cell growth, division and TJ regulation, remains down-regulated at the intestinal level in patients on a GFD [14]. These observations suggest a role for cellular structural alterations in the pathogenesis of CD. Of note, gliadin peptides, including P31–43, can interfere with actin rearrangements in both the CD mucosa and epithelial cell lines [9,15,16,17,18,19,20].

More recent studies predicted a role in CD pathogenesis for lipoma-preferred partner (LPP), C1ORF106 (C1 Orfan 106), Rho GTPase-activating protein 31 (ARHGAP31) and protein tyrosine phosphatase receptor type K (PTPRK) genes, which play a role in actin cytoskeleton rearrangement, cell–cell adhesion and in epidermal growth factor (EGF)/ EGF receptor (EGFR) pathway activation [21,22,23,24]. Transcripts of ARHGAP31, a Rho-GTPase-activating protein (GAP) which is required for cell spreading, polarized lamellipodia formation and cell migration, are altered in CD immune cells [24]. The Rho GTPases control the organization of the cytoskeleton, cell migration, cell–cell and cell–matrix adhesion, cell cycle progression, gene expression and cell polarity. Interestingly, RhoB was found to be involved in the intestinal remodeling of CD intestinal mucosa [25] and genetic variations of miosin IX B (MYO9B), which serves as a regulator of the Rho-dependent signaling pathways, have been associated with CD [26]. The link between these genes and a cellular phenotype has been studied only for LPP. In fact, alterations of LPP sub-cellular distribution, together with modifications of cell shape, the actin cytoskeleton and focal adhesions, have been described in fibroblasts from CD patients [27].

These data confirmed that structural cellular alterations, mainly involving the actin cytoskeleton, are present in cells from CD patients. Whether similar structural alterations can be observed also in circulating immune cells from CD patients remains to be defined.

In this study, we aimed to address this issue by investigating cell shape and the related cytoskeleton alterations in monocyte-derived DCs from children with CD compared with controls. The choice fell on peripheral blood monocyte-derived DCs since they are disjoined by the intestinal inflammatory milieu and are easily accessible, yet representing a cell subset of key immunological relevance in CD. To verify the hypothesis that structural cytoskeleton alterations are present in peripheral immune cells from CD patients, we studied the morphological characteristic of DC shape upon adhesion on fibronectin using three distinct assays and investigated their link with RhoA activity. We showed that DCs from CD patients presented lower levels of ARHGAP31, increased Rho A activation and displayed an elongated morphology when in contact with fibronectin compared with controls. Thus, adhesion on fibronectin revealed a “CD cellular phenotype” of DCs independent of gluten or HLA-DQ2 and regulated by Rho A activity.

## 2. Results

### 2.1. Rho-GTP and ARHGAP Levels in CD DCs

**Figure 1:**
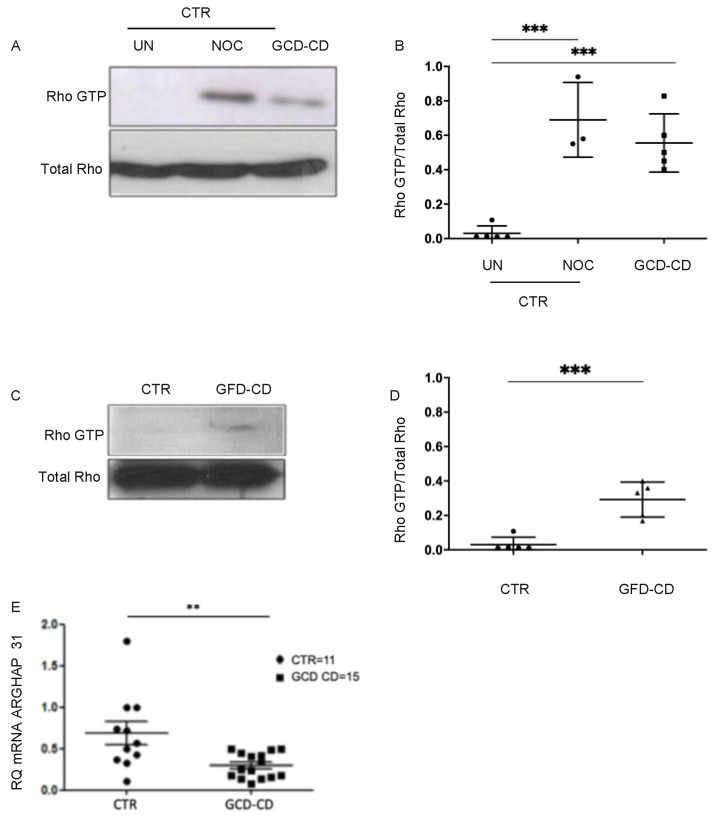
Rho-GTP and Rho GTPase-activating protein (ARHGAP) levels in dendritic cells (DCs) from celiac disease (CD) patients and controls. Ras homolog family member A (RhoA)-GTP levels are higher in DCs from CD patients than controls (CTR) after seeding on fibronectin for 1 h. 10^6^ DCs were plated for each subject and each condition. (**A**). Representative Western blot (WB) of RhoA activity (RhoA)-GTP levels in dendritic cells (DCs) from one control (CTR) and one celiac patient on a gluten-containing diet (GCD-CD). DCs from CTR were analyzed before (untreated, UN) and after treatment with nocodazole (NOC). (**B**). Densitometric analysis of WB performed as in A from five CTRs and five GCD-CD. NOC was used as a positive control on DCs from three CTRs. Compared with UN DCs from CTRs, NOC promoted RhoA activation, reaching similar levels to those observed in GCD-CD. Horizontal lines represent the mean; bars, the standard deviation. Ordinary one-way ANOVA with Tukey’s multiple comparison correction was performed; *** *p* < 0.001. (**C**). Representative WB showing RhoA-GTP levels in DCs from a CTR and a gluten-free diet (GFD-CD) patient. (**D**). Densitometric analysis of WB performed as in C from five subjects for each group. Horizontal lines represent the mean; bars, the standard deviation. Student’s *t*-test; * *p* < 0.05, ** *p* < 0.01. (**E**). Quantitative PCR analysis of Rho GTPase-activating protein 31 (ARHGAP31) mRNA levels showing decreased ARHGAP31 in GCD-CD DCs with respect to CTR. The number of subjects analyzed is indicated. The horizontal lines represent the median; bars, the standard deviation. Student’s *t*-test; ** *p* < 0.01.

DCs were generated from peripheral blood monocytes [28] of pediatric celiac patients on a gluten-containing diet (GCD-CD) in the active phase of the disease and on GFD (GFD-CD) in the remission phase of the disease, as well as those of healthy controls. Small GTP-binding proteins of the Rho family are key regulators of cytoskeletal dynamic and affect many cellular processes, including cell polarity, migration, vesicle trafficking and cytokine production. The Rho family of GTPases in particular has been shown to regulate cell shape and lamellipodia production in blood cells [29,30]. This prompted us to test RhoA activity in DCs from GCD-CD patients and controls when plated on fibronectin, a physiological substrate. In Figure 1A,B and Supplementary Figure S1A,B, Western blot analysis of RhoA-GTP, the activated form of RhoA, is shown. RhoA-GTP levels were higher in GCD-CD DCs with respect to controls. In fact, the ratio between the active form of Rho and the total protein (RhoA-GTP/Rho) was significantly lower in controls (0.03 ± 0.04) compared with GCD-CD patients (0.56 ± 0.17, *p* = 0.0001), indicating that in active CD patients, an activation of RhoA occurred in DCs upon contact with the substrate fibronectin (Figure 1B). Nocodazole, an inductor of RhoA activity, was used as a positive control to enhance RhoA-GTP levels in control DCs (0.69 ± 0.022 vs. 0.03 ± 0.04, *p* = 0.0004). Interestingly, a difference in RhoA-GTP levels was also found in DCs from GFD-CD (0.29 ± 0.1 vs. 0.03 ± 0.04 *p* = 0.0007) compared with controls (Figure 1C,D and Supplementary Figure S1C,D), suggesting that RhoA activation was independent of disease activity.

We also evaluated transcriptional levels of ARHGAP31, a Rho-GTPase activating protein, in DCs from GCD-CD patients (Figure 1E), showing a significant decrease in ARHGAP31 mRNA in GCD-CD compared with controls’ DCs (*p* < 0.01). The reduction of a negative regulator of Rho is in line with the increase in Rho activity; together, these data support the induction of the RhoA pathway in peripheral monocyte-derived DCs of CD patients.

### 2.2. DC Shape in CD Patients Shows a Different Shape Compared with Controls When Adhering on Fibronectin

**Figure 2:**
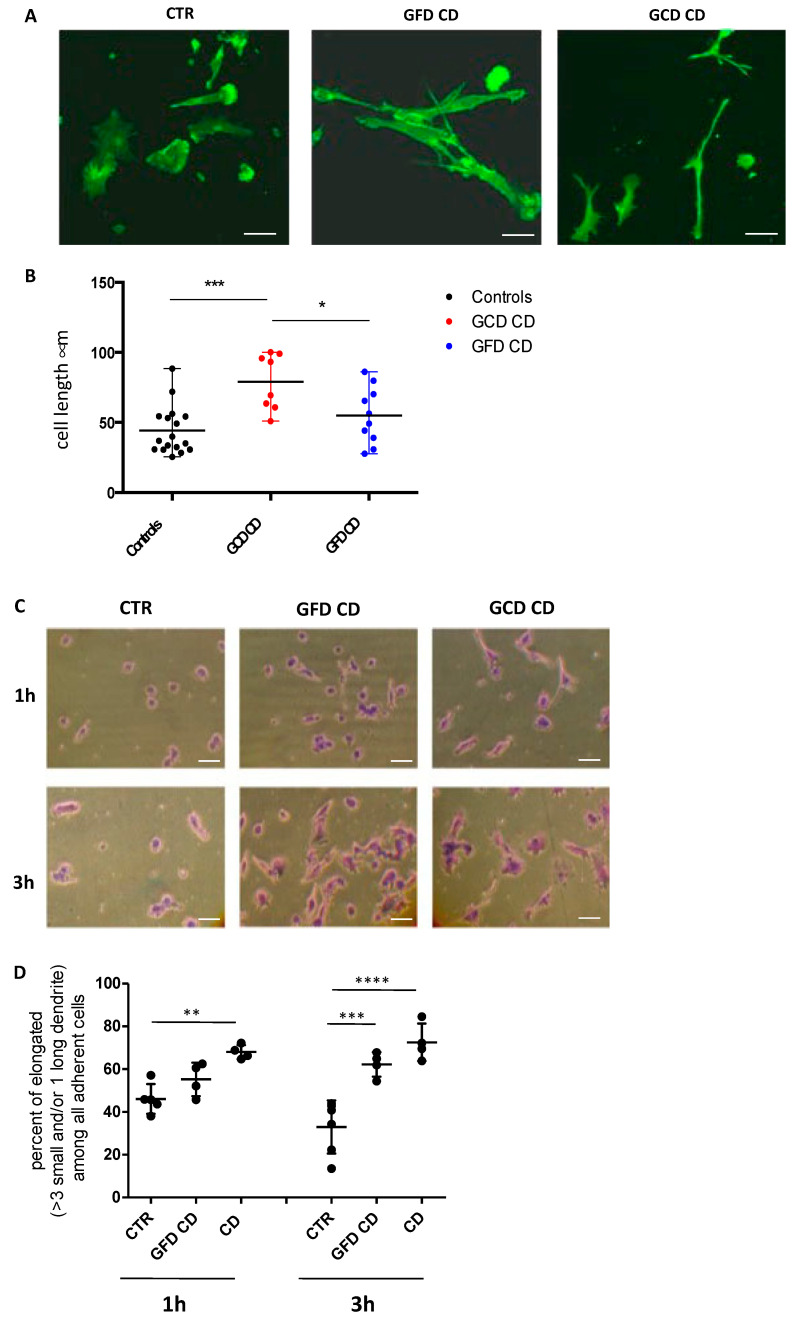
Elongated shape of DCs from CD patients and controls after spreading on fibronectin. (**A**) Representative images of fluorescein isothiocyanate (FITC)-conjugated phalloidin staining highlighting the actin cytoskeleton of DCs from CTR, GFD and GCD CD subjects. DCs were seeded on coverslips pre-coated with fibronectin at a concentration of 2.5 × 10^5^ cells/mL. Experiments were run in duplicate. Five random pictures from each duplicate were evaluated. Scale bar = 7 µm. (**B**) The maximum stretch of at least 20 DC per patients was measured and the average extension has been calculated and plotted. Quantitative analysis of DCs length, from 17 controls, 8 GCD-CD patients and 10 GFD-CD patients, after seeding on fibronectin for 3 h. Horizontal lines represent the mean; bars, the range. Ordinary one-way ANOVA with Tukey’s multiple comparison correction; * *p* < 0.05, *** *p* < 0.001. (**C**) Light microscopy images of fibronectin-adhering DCs from CTR, GFD-CD and GCD-CD patients spread on fibronectin for l (upper panel) and 3 h (lower panel). Crystal violet staining was performed. Scale bar = 10 µm. Each experiment was performed in duplicate, with 10^5^ DCs plated in each well. At least two independent experiments for each subject were performed. Six pictures were taken for each duplicate. Representative images are shown. (**D**) The percentage of fibronectin-adhering DCs showing an elongated shape, defined as a cell with more than three short and/or one long dendrites, among all spreading cells was assessed in five CTR, four GFD-CD and four GCD-CD. At least 50 cells per subject were counted. Scale bar = 10 µm. The horizontal lines represent the mean; bars, the standard deviation. Ordinary one-way ANOVA with Sidak’s multiple comparison test; ** *p* < 0.01, *** *p* < 0.001, **** *p* < 0.0001.

The increase in Rho activity in CD patients’ DCs prompted us to investigate the shape of DCs from GCD-CD and GFD-CD patients in comparison with controls. To highlight the morphology and actin cytoskeleton of DCs upon interaction with fibronectin, fluorescein isothiocyanate (FITC)-conjugated phalloidin immunofluorescent staining was performed (Figure 2A). DCs from controls, GCD-CD and GFD-CD patients were seeded on coverslips pre-coated with fibronectin and incubated. Phalloidin staining revealed differences in the shape of DCs from active CD patients with more numerous and long dendrites compared with controls. To quantify the differences in morphology, we measured the length of the phalloidin-stained DCs using the Zeiss LSM 510 software. The maximum stretch of at least 20 DCs per patients was measured, and the average extension was calculated and plotted (Figure 1B). DCs from GCD-CD patients (79.12 ± 19.96 µm) were significantly longer than control DCs (44.20 ± 17.21 µm; *p* = 0.0004). GFD-CD DCs also showed an elongated shape (54.88 ± 20.17 µm), although not statistically significant with respect to the controls’ DCs.

To further evaluate the altered morphology of DCs, we stained them with crystal violet and observed them with a transmitted light microscope after l hour (1 h) and 3 h adhesion on fibronectin (Figure 2C). DCs from CD patients showed a different shape upon fibronectin interaction when compared with controls. DCs showing more than three small dendrites and/or one long dendrite were considered to have an elongated shape. Dendrites were defined as “small” when they were shorter than half of the cellular body length, and “long” when they were longer than half of the cellular body length. Adhering cells were pictured and counted. The percentage of elongated DCs among adhering cells was evaluated and CD patients were found to have a statistically significant higher percentage of elongated DCs compared with controls (Figure 2D). After 3 h seeding on fibronectin, 71% ± 6.7 DCs from GCD-CD patients and 57% ± 6.6 DCs from GFD-CD patients showed an elongated shape compared with 36.95% ± 12.4 in the control group (*p* < 0.001, *p* < 0.01, respectively,). After l h seeding on fibronectin, only DCs from active CD patients showed a significantly more elongated shape compared with controls (*p* < 0.001).

### 2.3. DC Shape in Patients with Food Allergies or Inflammatory Bowel Diseases and Healthy Subjects Evaluated by Immunofluorescence Staining

**Figure 3:**
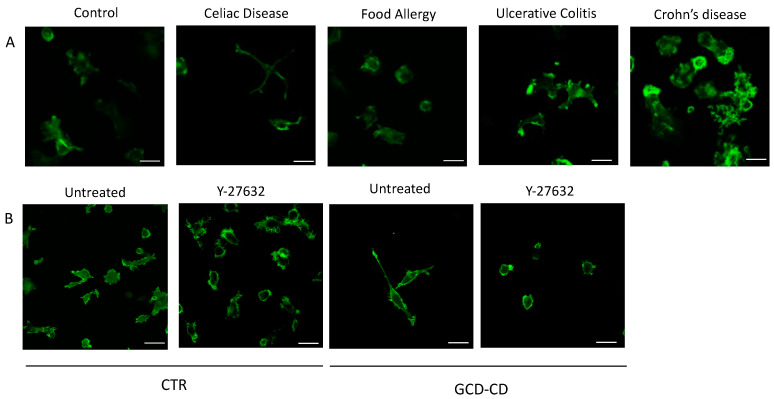
DC shape and cytoskeleton evaluated by immunofluorescence staining. (**A**) Representative immunofluorescence images of FITC-conjugated phalloidin staining, highlighting the actin cytoskeleton of fibronectin-adhering DCs from one control and patients with GCD-CD, food allergy or inflammatory bowel disease (ulcerative colitis and Crohn’s disease), showing that the elongated morphology of DCs was specific to CD. FITC-conjugated phalloidin staining was performed on DCs plated on coverslips pre-coated with fibronectin at a concentration of 2.5 × 10^5^ cells/mL. One representative image out of at least three subjects per group is shown. Each experiment was done in duplicate; six pictures were taken for each duplicate. Scale bar = 7 µm. (**B**) Representative immunofluorescence images of phalloidin staining of DCs from four GCD-CD patients and controls plated on coverslips pre-coated with fibronectin at a concentration of 2.5 × 10^5^ cells/mL and incubated at 37 °C for 3 h before (Untreated) and after treatment with the Y-27632 Rho-associated protein kinase (ROCK) inhibitor. Treatment with Y-27632 prevented protrusion formation and cell shape alterations observed in GCD-CD DCs (right panels), confirming that the DC shape of CD patients depended on RhoA activation.

To assess whether the elongated morphology described in DCs from CD patients was also present in other diseases and to explore whether intestinal inflammation could contribute to altering DC shape, we evaluated the morphology and actin cytoskeleton of DCs from patients with inflammatory bowel diseases and food allergies. DCs from patients with Crohn’s disease, ulcerative colitis and food allergies, were seeded on coverslips pre-coated with fibronectin and analyzed by confocal microscopy upon a FITC-conjugated phalloidin immunofluorescent staining to highlight the actin cytoskeleton (Figure 3A). None of the analyzed patients affected by diseases other than CD showed the specific elongated morphology observed in CD DCs, suggesting that the observed morphological changes are specific to CD. Cytoskeleton alterations characterized by increased membrane ruffling (Figure 3A) have been described in DCs from Crohn’s disease patients and the related to inflammation [31].

Importantly, the inhibition of RhoA activation by treatment with the Rho-associated protein kinase (ROCK) inhibitor Y-27632 was able to prevent DC shape alterations in CD (Figure 3B), demonstrating that the elongated shape of DCs from CD patients was dependent on RhoA activity.

Moreover, to exclude the possibility that HLA-DQ2-predisposing alleles could impact the observed morphology of DCs in CD patients, we investigated DC shape in control subjects carrying or not carrying the HLA-DQ2 haplotype and did not observe any difference (Supplementary Figure S3).

### 2.4. DC Shape in CD Patients and Healthy Subjects Evaluated by Video Time-Lapse Microscopy

**Figure 4:**
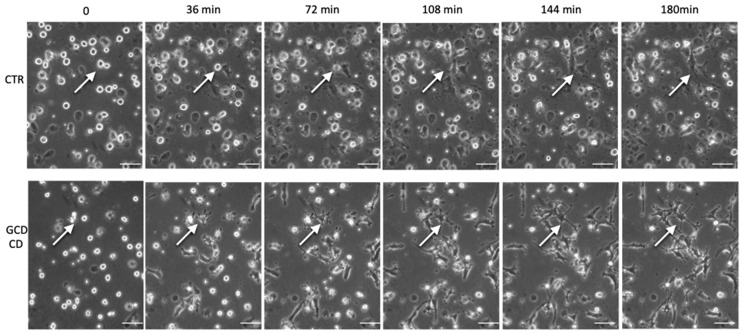
Spreading ability of fibronectin-adhering DCs. DCs from three active celiac patients (GCD-CD) and from three controls were seeded on wells pre-coated with fibronectin at a density of 2.5 × 10^5^ cells/mL, and their spreading properties were recorded live for 3 h to observe dendrite formation. Fifty cells in each frame were followed over time for a total of 200 DCs per patient. One picture every 36 min of the movie is shown, starting from T0 (the time when DCs were seeded in the well) until T180 (the time when the film was stopped). Images from a representative CD patient and a representative control out of at least three subjects per group are shown. White arrows indicate the same cells in each panel. Scale bar = 20 µm.

Monocytes derived DCs from active CD patients and healthy subjects were plated on fibronectin-coated wells and filmed live for 3 h by video time-lapse microscopy to observe their ability to interact with the substrate (see Supplemental Movies S1 and S2). DCs were recorded while spreading on fibronectin. Before spreading, DCs appeared as small translucent circles, while upon spreading on the substrate, they appeared less translucent and started to project dendrites. Within 2 h, dendrites were formed and could retreat. DCs from CD patients showed longer dendrites with respect to the controls at any time observed. Images were taken every 18 min. In Figure 4, images taken every 36 min from two representative subjects are shown. The ability of the DCs from CD subjects to adhere on the substrate was not impaired; in fact, the number of adhering cells after seeding on fibronectin was the same as in the control (Supplementary Figure S2).

### 2.5. Evaluation of DC Markers of Maturation in Controls and CD Patients before and after Adhesion on Fibronectin

**Figure 5:**
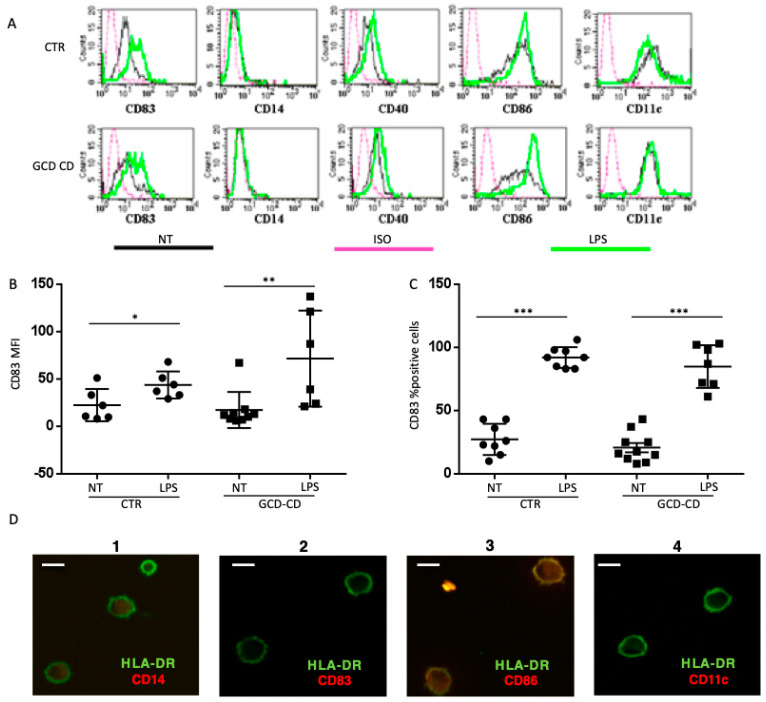
DCs’ surface marker profile of CD patients and controls before and after adhesion on fibronectin. (**A**). Flow cytometric analysis of DCs’ surface markers profile before interaction with fibronectin. DCs generated from peripheral monocytes were used at a concentration of 2.7–8.3 × 105 cells/mL in 24-well plates and stained with the antibodies indicated in the figure. Fluorescence intensity of the analyzed samples is represented in a histogram plot with a black line (NT, not treated); green lines refer to lipopolysaccharide (LPS)-treated samples; pink lines refer to the isotype-matched control (ISO). Representative histogram plots from one GCD-CD patient and one CTR are shown. (**B**,**C**). Quantification and statistical analysis of cluster differentiation (CD)83 surface expression as assessed by flow cytometry in DCs from CD patients and CTR showing similar CD83 surface expression levels. For CD83, mean fluorescence intensity (MFI) (**B**) and the percentage of positive cells (**C**) are shown. Overnight LPS treatment, used as a positive control, induced a significant increase in CD83 MFI (**B**) and the percentage of CD83+ cells (**C**) in both CTR and in CD subjects compared with non-treated (NT) cells. Each dot represents one subject. The horizontal lines represent the median; bars, the standard deviation. Student’s *t*-test; * *p* < 0.05, ** *p* < 0.01, *** *p* < 0.001. (**D**). DCs from active CD patients were plated onto coverslips pre-coated with fibronectin at a concentration of 2.5 × 105 cells/mL and incubated at 37 °C. To assess the surface marker profile after 3 h of adhesion on fibronectin by confocal microscopy, DCs were stained with FITC-conjugated anti-human leukocyte antigen (HLA)-DR antibody (green staining in each image) and with a phycoerythrin (PE)-conjugated antibody (red staining) against different surface markers, as indicated in each image. Yellow color represents merged staining. Cells appear round because of the four-degree incubation used to avoid antibody endocytosis. Representative images of at least three subjects done in duplicate are shown. More than 20 cells per patients were observed. Scale bar = 8 µm.

To evaluate whether the different DC shape observed in CD subjects was dependent on immunological activation of peripheral cells, the immunological surface profile of DCs before and after interaction with fibronectin was assessed. Surface expression of cluster of differentiation (CD)14, CD40, CD83, CD86 and CD11c was evaluated by flow cytometry to establish the DC phenotype before interaction with fibronectin (Figure 5A,B). CD14 staining was performed to exclude monocyte contamination; CD86, CD83 and CD40 expression was assessed to determine DCs’ immunological activation and maturation (CD83), while CD11c staining ensured that DCs belonged to the myeloid subset. Notably, DCs from both healthy subjects and CD patients had similar surface marker profiles (Figure 5A,B), consistent with immature (CD83^–^) non-activated (CD40^–^, CD86^low^) and myeloid DCs (CD14^–^CD11c^+^).

Overnight lipopolysaccharide (LPS) induced maturation of DCs both in controls and in CD subjects (Figure 5A–C), as shown by a statistically significant increase in both CD83 mean fluorescence intensity (Figure 5B) and the percentage of positive cells (Figure 5C) upon LPS treatment compared with untreated cells. (LPS).

The ability of fibronectin interaction to induce an alteration of DCs’ surface marker profile was also tested. Double immunofluorescence staining was performed on DCs from active CD patients and healthy controls after adhesion on fibronectin, showing that adhesion on fibronectin did not alter the surface marker profile (HLA-DR^+^, CD14^+^ CD83^+^, CD86^+^, CD11c^+^) of DCs from CD patients (Figure 5D). Altogether, these data show that, DCs from both controls and CD patients displayed an immature/adhering phenotype in the presence of fibronectin; however, DCs from CD patients had a more elongated shape.

## 3. Discussion

In this study, we analyzed the morphology and actin rearrangements of monocyte-derived DCs of pediatric CD patients, using three different techniques, namely immunofluorescence, transmitted light microscopy upon crystal violet staining and video time-lapse microscopy, showing the presence of structural alterations of CD cells, defining a CD-specific cellular phenotype. Importantly, we linked this phenotype to increased RhoA activity and to decreased ARHGAP transcriptional levels, a Rho-GAP that genetic studies have found to be associated with CD pathogenesis.

Alterations of the actin cytoskeleton and tight junctions have previously been described in CD enterocytes, indicating that cytoskeletal rearrangements and cell contacts might be involved in the CD lesions [5,6,7,8,9,10,11,12]. An increase in intestinal permeability resulting from a loss of the barrier function due to tissue damage is a constant feature in CD subjects [32]. GFD can partially or fully restore these alterations [11,33].

In this paper, we show an alteration of the morphology and actin cytoskeleton of DCs in pediatric CD patients. Adhesion on fibronectin induced the formation of elongated dendrites in a higher number of DCs from CD patients with respect to controls, indicating that CD DCs reacted differently upon interaction with a physiological substrate. This altered shape upon contact with the substrate was displayed by DCs from both treated and untreated pediatric CD patients, revealing a “CD cellular phenotype” independent of dietary gluten content. This phenotype is specific to children with CD, as it was absent in DCs from pediatric patients with Crohn’s disease, ulcerative colitis and food allergies. Moreover, this CD-specific phenotype does not depend on the disease activity, since it could also be observed in children on a GFD and did not seem to depend on the presence of predisposing HLA-DQ2 alleles, since it was not observed in controls carrying this haplotype. Furthermore, DCs from both CD pediatric patients and controls had the same surface immunological profile before and after adhesion on fibronectin, corresponding to an immature, non-activated and adhering phenotype of myeloid DCs. A similar CD cellular phenotype characterized by altered cell shape and actin distribution has also been found in skin fibroblasts from CD patients, another cell population located far from the intestinal lesion and in the absence of gliadin peptides [27], suggesting a structural constitutive alteration.

In this paper, we also investigated the differences in the activity of RhoA, a protein able to regulate cell shape and dendrite formation in monocytes [34]. Interestingly, we found that in comparison with controls, upon contact with fibronectin, RhoA-GTP levels were increased in DCs from pediatric CD patients and that the inhibition of RhoA activity prevented the morphological alterations resulting in an elongated shape observed in DCs from CD patients. Altogether, these data point to an alteration of the RhoA pathway in CD DCs. Accordingly, together with an increased RhoA activity, we observed a decrease in ARHGAP31 transcript levels. The reduction of a negative regulator of Rho is, in fact, compatible with increased Rho activity. In line with our findings, genetic studies on peripheral blood monocytes point to an altered expression of ARHGAP31 in CD. This observation could suggest that the CD cellular phenotype described in this study could be associated with a “cell type-specific expression” of CD-related genes, including ARHGAP31 [24].

Constitutive alterations of CD cells, in the absence of a gliadin challenge, have been described in different systems and both in vivo and in vitro assays [35,36,37,38]. These alterations point to a derangement of signaling, proliferation and the cytoskeleton, leading to inflammation, innate immunity activation and cellular stress in the enterocytes and fibroblasts of CD patients. An impairment of the endocytic trafficking could contribute to explaining these structural alterations [36]. More recently, ex vivo experiments in intestinal organoids [39,40] demonstrated that staminality and inflammation pathways are altered in GFD-CD organoids even before gluten exposure. Moreover, gluten challenge further impacts the same pathways already altered in GFD biopsies [37] and organoids [39].

Taken together, these observations describe a “CD cellular phenotype” characterized by altered cell shape, actin remodeling, signaling and innate immunity activation, suggesting that even in polygenic disorders, a “disease-specific cellular phenotype” can be observed in almost all pediatric subjects. Further studies will clarify whether these same alterations can be observed in adults with CD. Similarly, a disease-specific cellular phenotype, independent of disease-associated gene variants, has been observed in another polygenic intestinal inflammatory diseases, such as Crohn’s disease. Indeed, Paneth cells from Crohn’s intestinal biopsies display an impairment in autophagy regardless of the presence of genetic variants of the autophagy pathway, which have been detected only in few Crohn’s patients [41].

In conclusion, we showed that adhesion on fibronectin revealed a different morphology of DCs from pediatric CD patients compared with controls. This altered DC shape may be considered as part of the gluten-independent alterations of CD cells defining a “CD cellular phenotype”, characterized not only by alterations of the signaling and inflammatory markers, but also by structural cellular alterations [21,36,42,43,44].

## 4. Materials and Methods

### 4.1. Patients

Monocyte-derived DCs were obtained from pediatric CD patients on a GCD in the active phase of the disease (37 patients, mean age 6.6 years), or on a GFD in the remission phase of the disease (11 subjects, mean age of 11 years) and from controls, including subjects affected by gastroesophageal reflux (31 subjects, mean age of 7.5 years). GCD-CD patients included children with positive serology, including anti-tTg immunoglobulin A (IgA) antibodies (>50 U/mL) and anti-endomysium antibodies (EMA), and displaying duodenal villous atrophy (Marsh T3) at the small intestinal biopsy (Table 1). Patients with GFD-CD had negative CD-specific serology, including anti-tTg IgA antibody titers ranging between 0 and 1.5 U/mL, negative EMA and a normal duodenal biopsy (Marsh score T0). Patients had been on a GFD for at least 4 years prior to enrollment; the adherence to the GFD was complete and patients had remission of the symptoms and negative CD-specific serology. Anti-tTg antibody titers were measured using the Eurospital kit EU-tTG (Trieste, Italy). To compare our observations with patients with other intestinal inflammatory conditions, we also enrolled four pediatric patients with food allergies associated with intestinal symptoms and 6 with pediatric inflammatory bowel diseases, particularly 3 subjects with Crohn’s disease and 3 with ulcerative colitis. All enrolled subjects were children and their parents provided written informed consent for the use of their blood cells in this study. The protocol for this study was approved by the Ethical Committee of the University “Federico II”, Naples, Italy (Ethical Committee approval, Protocol no. 230/05).

### 4.2. Determination of HLA-DQ2 and -DQ8

The determination of HLA-DQ2 and/or -DQ8 of control subjects was performed using a commercial kit (Eu-DQkit, Eurospital, Trieste, Italy). DNA was extracted from whole blood and subjected to 2 different PCR reactions. A specific genomic sequence encoding for human globin (268 bp) was used as a control, according to the manufacturers’ protocol.

### 4.3. Cell Culture

Peripheral blood mononuclear cells (PBMCs) were isolated by density gradient centrifugation of heparinized blood using Lymphocyte Separation medium (Lonza, Walkersville, MD, USA). PBMCs were cultured in RPMI-1640 (Invitrogen, San Giuliano Milanese, Italy) in 6-well plates (Corning, Euroclone, Milano, Italy) at a density of 2.5 × 10^6^/mL at 37 °C in a 5% CO_2_ humidified atmosphere. After l h, non-adherent cells were removed. Adherent cells were cultured in RPMI-1640 supplemented with 10% fetal bovine serum (FBS) (GIBCO, Thermo Fisher, Milano, Italy), 1% L-glutamate (ICN), 100 U/mL streptomycin or penicillin, and 80 µL fungizone (GIBCO, Thermo Fisher, Milano, Italy). To obtain DCs, 1000 U/mL of granulocyte-monocyte colony stimulating factor (GM-CSF, RD System, Minneapolis, Minnesota, USA) and 800 U/mL of interleukin 4 (IL-4, RD System, Minneapolis, Minnesota, USA) were added to the culture medium on Day zero and Day 3. Monocytes differentiated into immature DCs during 6 days of culture. DCs were harvested at Day 6 or 7.

### 4.4. Rho GTP Assay

Rho activation [28] was tested by the EZ-Detect Rho Activation kit (Pierce, Rockford, IL, USA) following the manufacturers’ instructions. In brief, 1 × 10^6^ DCs obtained from 5 GCD-CD, 5 GFD-CD and 5 control subjects were seeded on fibronectin for l h and then washed with ice-cold Tris-buffered solution pH 7.5. They were then lysed in Lysis/Binding/Wash Buffer (25 mM Tris-HCl, pH 7.5, 150 mM NaCl, 5 mM MgCl_2_, 1% Nonidet P-40 (NP-40), 1 mM Dithiothreitol (DTT), 5% glycerol) with 1 pg/mL each of leupeptin and aprotinin, and 1 mM phenylmethylsulfonyl fluoride (PMSF) and incubated on ice for 5 min (all from Sigma-Aldrich, Milan, Italy). Cell lysates were clarified by centrifugation at 16,000× *g* at 4 °C for 15 min. Glutathione S-transferase (GST) Rhotekin Rho binding domain (RBD) (400 µg) was added to the spin cup containing the glutathione resin and then supernatants were incubated with GST-Rhotekin-RBD beads for 1 h at 4 °C. Beads were then washed 3 times with a cold lysis buffer. Bound GTP-RhoA was detected by Western blotting using monoclonal anti-RhoA antibodies (1:1000) [28,29]. All performed following manufacturer instructions (Thermo Fisher Scientific, Milan, Italy). Densitometry analysis was performed using Image J (https://imagej.nih.gov/ij) (accessed on 3 February 2021). The amount of GST-Rhotekin-RBD-bound RhoA was normalized to the total amount of RhoA in cell lysates and expressed as the GTP-RhoA/RhoA ratio. DCs from 3 CTR were treated with Nocodazole (Sigma-Aldrich, Milan, Italy) (20 µM) as a positive control [29].

### 4.5. PCR

Total RNA was extracted from DCs from patients with CD on a GFD and CTR using TRIZOL reagent (Ambion Life Technologies, Monza, Italy). The mRNA concentration was measured using a Nanodrop spectrophotometer, and the RNA quality was analyzed using agarose gel electrophoresis in Tris/Borate/Ethylenediaminetetraacetic acid (EDTA) buffer (TBE, Sigma, Milan, Italy). The RNA (1 µg) was reverse transcribed into cDNAs using the High Capacity cDNA Reverse Transcription kit (Applied Biosystems, Monza, Italy) according to the manufacturer’s protocol. Experiments were performed with approximately 40 ng of cDNA templates, according to the manufacturer’s protocol (TaqMan Gene Expression Assay), using a 7900 HT Fast Real-Time PCR system. The gene expression assay used for the ARHGAP gene was Hs00929215_m1 (Life Technologies, Monza, Italy), and the probe was located on Chromosome 3 (Chr3) exons 11–12. The expression of each gene was normalized to the expression of an endogenous housekeeping gene (Beta actin). Relative quantification was performed using the ΔΔCt method. SDS software (ABI, version 1.4 or 2.4) was used to analyze the raw data.

### 4.6. DC Spreading on Fibronectin: Actin Assay

Sterile 12-mm glass coverslips (Knittel Glaser, Braunschweig Germany) were pre-coated overnight with 5 μg/mL fibronectin, then blocked for 1 h with 5% bovine serum albumin (BSA). DCs (obtained as described above) were plated onto fibronectin-coated coverslips in phosphate buffer solution (PBS) containing 5 mg/mL fetal bovine serum (FBS), 5 mM glucose and 0.3 MgCl_2_ at a concentration of 2.5 × 10^5^ cells/mL, and incubated at 37 °C to evaluate their ability to spread onto a physiological substrate. After 3 h of adhering, cells were fixed in 3% paraformaldehyde (PFA, Sigma-Aldrich, Milan, Italy) and permeabilized with 0.2% Triton X-100 (Biorad, Milan, Italy). Actin rearrangements were revealed by staining with 10 μg/mL FITC-conjugated phalloidin or 0.1 mg/mL Tex-red phalloidin (Sigma-Aldrich, Milan, Italy) for 45 minutes before mounting with Mowiol (Sigma-Aldrich, Milan, Italy) (35–37). Images were acquired and processed with KS300 software (Carl Zeiss MicroImaging, Inc., Jena, Germany) [24]. Experiments were run in duplicate. Five random pictures from each duplicate were evaluated. For each subject, at least 20 adhering cells were analyzed to measure cell length.

### 4.7. DC Spreading on Fibronectin: Crystal Violet Assay

First, 96-well plates (Nunc, Maxisorp, VWR International, Milan, Italy) were pre-coated with 50 μg/mL fibronectin in sterile water and left overnight at 4 °C. Wells were washed twice with sterile PBS and blocked with 5% BSA in PBS for 1 h at room temperature. All the experiments were done in duplex. After blocking, wells were washed twice before plating 10,000 DCs/well in PBS containing 5 mg/mL BSA, 5 mM glucose and 0.3 mM MgCl_2_, and incubated at 37 °C for 1 h and 3 h. Upon incubation at 37 °C, DCs were washed and stained using a solution containing 79.5% PBS, 20% methanol and 0.5% crystal violet (all from Sigma-Aldrich, Milan, Italy). The following day, wells were washed, fibronectin adhering DCs were observed, 6 random pictures were taken from each duplicate and at least 50 cells adhering cells in each picture were counted. In particular, DCs showing more than 3 small dendrites and/or 1 long dendrite were considered to have an elongated shape. For small dendrites, we defined this as a dendrite shorter than the length of the cell body, while long dendrites were longer than the cell’s body. At least 4 independent experiments were performed, and the average was calculated from 24 quantified fields.

### 4.8. Time-Lapse Experiments

DCs were plated onto with wells pre-coated with fibronectin at a concentration of 2.5 × 10^5^ cells/mL and kept in a specially designed incubator (OXO-lab, Naples, Italy) monitoring the temperature and CO_2_ to allow live observation. More than 200 DCs in each well were filmed for 3 h; sets of frames were acquired every 18 min [41].

### 4.9. Flow Cytometric Analysis

DCs were harvested and re-suspended in complete Roswell Park Memorial Institute (RPMI) (GIBCO, Thermo Fisher, Milan, Italy) at a concentration of 2.7–8.3 × 10^5^ cells/mL in 24-well plates. After 1 h of incubation of DCs in absence or presence of lipopolysaccharide (LPS, Inbios, Naples, Italy) at 37 °C, cells were collected, washed twice and blocked for 10 min at 4 °C with human IgG. DCs were then incubated for 30 min at 4 °C with different fluorochrome-labeled antibodies (BD Bioscience, Milan, Italy), as indicated in the figure legends, diluted in Fluorescence-activated cell sorting (FACS)-PBS (BD Bioscience, Milan, Italy), washed twice using FACS-PBS and fixed with 2% paraformaldehyde (PFA) (Sigma-Aldrich, Milan, Italy). Unstained cells were used as negative control [42]. Stained cells were processed by FACS Calibur BD Pharmingen and analyzed by CellQuest (BD Biosciences, Milan, Italy).

### 4.10. Immunofluorescence Staining of DC Surface Markers

DCs were plated onto coverslips pre-coated with fibronectin at a concentration of 2.5 × 10^5^ cells/mL and incubated at 37 °C as described above. Surface staining was performed using a fluorescein isothiocyanate (FITC)-conjugated anti-HLA-DR to stain all DCs. Anti-CD14, anti-CD83, anti-CD86 and anti-CD11c phycoerythrin (PE)-conjugated antibodies (all from BD Biosciences Pharmigen, Milan, Italy) were used to perform the second staining. DCs were blocked for 10 min with human IgG at 4 °C, treated with the first antibody for 30 min, washed and stained with a second antibody for 30 min. Coverslips pre-coated with fibronectin were washed, fixed in 3% PFA, washed several times and mounted with Mowiol. Stained cells were observed with an Axioplan2 fluorescent microscope. Images were acquired and processed with KS300 software (Carl Zeiss MicroImaging, Inc., Jena, Germany) [24].

### 4.11. Statistics

GraphPad Prism 6 (GraphPad Software, San Diego, CA, USA) was used for statistical analysis and graphic representation. Statistical analyses of differences were performed using Student’s t-test or ordinary one-way ANOVA with Tukey’s multiple comparison correction, as indicated. A *p*-value < 0.05 was considered statistically significant [34].

## Figures and Tables

**Table 1:** Clinical data of patients and controls.

Patients	Mean Age (Years)	Sex	Biopsy Marsh Classification *	Serum Anti-TG2 Antibodies (U/mL)	Anti-Endomysium Antibodies (EMA)
Controls (N = 31)	7.5	M = 17 F = 14	T0	0–1.5	Negative
GCD-CD (N = 37)	6.6	M = 12 F = 25	T3a = 0 T3b/c = 16 T3c = 21	>50	Positive
GFD-CD (N = 11)	11.0	M = 4 F = 7	T0	0–1.5	Negative
Food Allergy (N = 4)	2.5	M = 2 F = 2	N/A	0–1.5	Negative
Ulcerative Colitis (N = 3)	7.5	M = 1 F = 2	N/A	0–1.5	Negative
Crohn’s Disease (N = 3)	6.6	M = 1 F = 2	T0	0–1.5	Negative

* T0: Normal; T3: flat destructive lesion (a: mild, b: moderate, c: total); ** N/A: non-applicable.

## Supplementary Materials

Supplementary materials can be found at https://www.mdpi.com/1422-0067/22/5/2708/s1.

## Abbreviations

ARHGAP31	Rho GTPase-activating protein 31
BSA	bovine serum albumin
CD (-83, -14, -40, -86, -11c)	cluster of differentiation
CD	celiac disease
C1ORF108	C1 Orfan gene 108
DCs	dendritic cells
EGF	epidermal growth factor
EGFR	epidermal growth factor receptor
FBS	fetal bovine serum
FITC	fluorescein isothiocyanate
GCD	gluten-containing diet
GFD	gluten-free diet
GTP	guanosine triphosphate
h	hour/hours
HLA	human leukocyte antigen
Ig	human immunoglobulin
LPP	lipoma-preferred partner
LPS	lipopolysaccharide
MAGI2	membrane-associated guanylate kinase, WW and PDZ domain-containing 2
MYO9B	myosin IXB
NOC	nocodazole
NT	not treated
PARD3	Par-3 family cell polarity regulator
PBMCs	peripheral blood mononuclear cells
mo-DCs	monocyte derived dendritic cells
PE	phycoerythrin
PFA	paraformaldehyde
PPP2R3A	protein phosphatase 2 regulatory subunit B’’ alpha
PTPRK	protein tyrosine phosphatase receptor type K
RhoA	Ras homolog family member A
ROCK	Rho-associated protein kinase
Th	T helper
TJ	tight junctions
tTg	tissue transglutaminase
