# Supplementary material for: Pediatric Celiac Disease Patients Show Alterations of Dendritic Cell Shape and Actin Rearrangement
Source: Int J Mol Sci. 2021 Mar 8;22(5):2708. doi: 10.3390/ijms22052708 (PMC7962447; doi:10.3390/ijms22052708)
Supplement: Supplementary file 1 [file ijms-22-02708-s001.zip › Supplementart materials.docx]

Alterations of celiac dendritic cell shape and actin rearrangement: a biomarker for

the disease.

Valentina Discepolo, Maria Leonarda Gertrude Ten Eikelder, Giuliana Lania, 1Merlin

Nanayakkara, Leandra Sepe, Riccardo Troncone, Salvatore Auricchio, Renata Auricchio

Giovanni Paolella, and Maria Vittoria Barone.


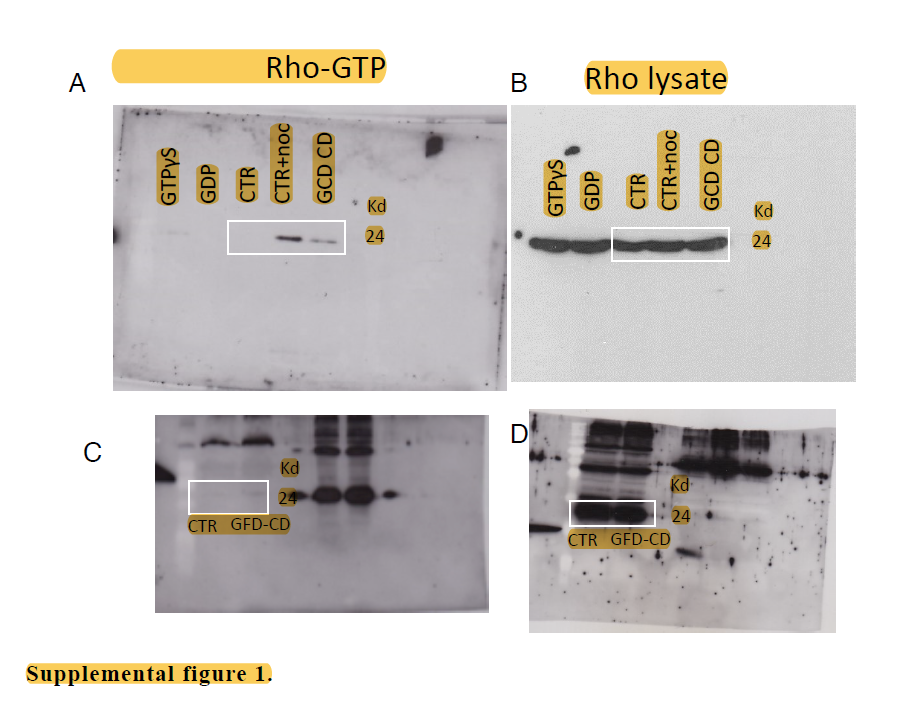


**Supplementary figure** l: Un-cropped Blots from figure 1. The letters (A, and C) on the un-cropped

gels refers to those of the figure 1.


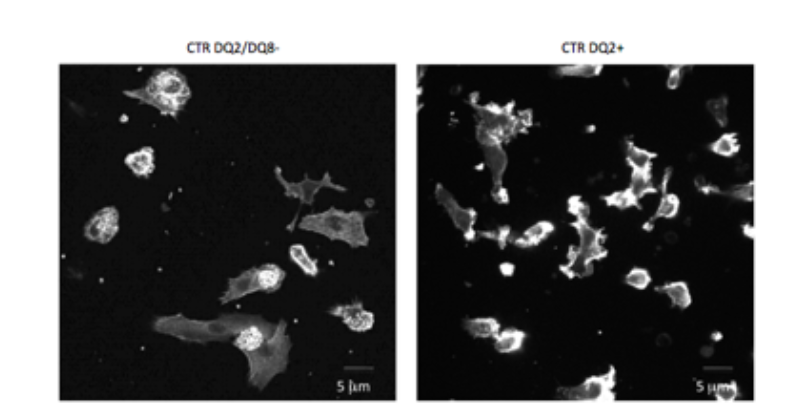


**Supplemental Figure** 2. DCs shape alterations did not depend on HLA-DQ2. Tex

red-conjugated phalloidin staining of DCs from HLA-DQ2 negative and DQ2 positive

controls after seeding on fibronectin for 3 h. Representative images of at least 3

subjects done in duplicate are shown. No differences in cell shape after contact with

fibronectine was detected.


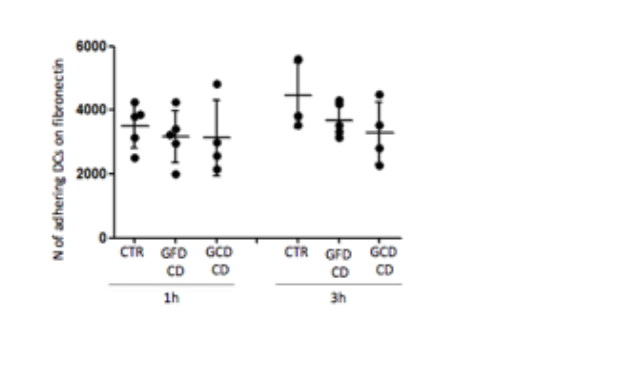


**Supplemental figure** 3: Adhesivety on fibronectin was similar in CTR and GCD-CD and GFD-CD

DCs. For the adesivity assay cells were detached by Versene solution (Versene 0.5mM; EDTA,

acido etilendiamminotetraacetico, in PBS, phosphate-buffered saline, 1x) treatment and seeded on

50 micrograms of fibronectin in resuspension solution (5mg/ml BSA, bovine serum albumin, 5mM

glucosio, 0.3mM MgCl2) and then left at 37?C for 30 minutes. After washing with PBS (2x), crystal

violet solution (cristal violet 0.5% in 20% metanol) was added for an ON at room temperature. All

reagents were from Sigma-Aldrich, Milan, Italy. Cells were washed with PBS (2x) and with

distilled H2O (1x) and then counted with a Microscope (Zeiss, Milan, Italy).
